# Psip1/Ledgf p75 restrains *Hox* gene expression by recruiting both trithorax and polycomb group proteins

**DOI:** 10.1093/nar/gku647

**Published:** 2014-07-23

**Authors:** Madapura M. Pradeepa, Graeme R. Grimes, Gillian C.A. Taylor, Heidi G. Sutherland, Wendy A. Bickmore

**Affiliations:** MRC Human Genetics Unit, MRC Institute of Genetics and Molecular Medicine at University of Edinburgh, Crewe Road, Edinburgh EH4 2XU, UK

## Abstract

Trithorax and polycomb group proteins are generally thought to antagonize one another. The trithorax family member MLL (myeloid/lymphoid or mixed-lineage leukemia) is presumed to activate *Hox* expression, counteracting polycomb-mediated repression. PC4 and SF2 interacting protein 1 (PSIP1)/p75, also known as LEDGF, whose PWWP domain binds to H3K36me3, interacts with MLL and tethers MLL fusion proteins to *HOXA9* in leukaemias. Here we show, unexpectedly, that Psip1/p75 regulates homeotic genes by recruiting not only MLL complexes, but also the polycomb group protein Bmi1. In *Psip1^−/−^* cells binding of Mll1/2, Bmi1 and the co-repressor Ctbp1 at Hox loci are all abrogated and *Hoxa* and *Hoxd* mRNA expression increased. Our data not only reveal a potential mechanism of action for Psip1 in the regulation of *Hox* genes but also suggest an unexpected interplay between proteins usually considered as transcriptional activators and repressors.

## INTRODUCTION

The Homeotic (*Hox*) gene family encodes transcription factors essential for patterning the anterior–posterior body axis. The developmental pattern of *Hox* gene expression is thought to be maintained by two groups of proteins: Polycomb repressive complexes (PRCs) maintain *Hox* genes in a silent state (1). The PRC2 complex contains the Ezh2/Ezh1 histone methyltransferases (HMTs) that mediate H3K27 trimethylation (H3K27me3) (2) and PRC1 contains the Bmi1 or Mel18/Ring1a/b heterodimer which can ubiquitinate H2AK119 and can compact chromatin (3). Trithorax (Trx) proteins (MLL proteins in mammals) of the COMPASS-like family have histone H3 lysine 4 (H3K4) methyl transferase activity and are generally thought to maintain the active expression level of *Hox* genes (4,5), and may function as anti-repressors to prevent the repressive function of polycomb (6–8).

Six mammalian COMPASS-like complexes have been identified, each with a SET domain-containing HMT subunit including; Set1A/KMT2F, Set1B/KMT2G and four MLL-family proteins—MLL1/KMT2A, Mll2/KMT2B, MLL3/KMT2C, MLL4/KMT2D (9,10). Each of these complexes associates with proteins that can modulate target site selection and enzymatic activity. Set1A/B are associated with Wdr82 (11,12), Mll1 and Mll2 are associated with Menin (5), Mll3 and Mll4 with PTIP (13).

Among the *MLL* genes, *MLL1* has been the most extensively studied as it is frequently involved in leukemia-associated chromosomal translocations, where its fusion to a variety of proteins is accompanied by dysregulated *Hox* expression in haematopoiesis (14). Mice mutant for Mll1, or for its Set domain, have homeotic transformations of the axial skeleton and aberrant *Hox* gene expression (15,16). In contrast to SET1A/B, Mll1 and Mll2 have few target genes, but these include *Hox* genes (17,18). However, the relationship between Mlls and H3K4 methylation is complex. Mll1 is dispensable for most of the H4K4me3 at *Hox* genes in fibroblasts (5) and in mouse embryonic stem cells (mESCs) (18). But H3K4me3 at some *Hox* promoters in mESCs requires Mll1, at others it requires Mll2, and at some it requires both. Many promoters do not require either Mll1 or Mll2 indicating that a third enzyme is responsible for H3K4 trimethylation on *Hox* genes (18). Indeed, recent *in vitro* evidence indicates that Trx, Mll1 and Mll2 catalyse H3K4 monomethylation rather than H3K4me3 (19).

In flies Trx complexes bind to specific response DNA elements (TREs). Mammalian TREs have not been identified and the mechanism of MLL recruitment to *Hox* genes is not clear. Two lncRNAs expressed from the *Hoxa* cluster, and linked to *Hox* gene activation, have been suggested to function through recruitment of the MLL1 complex. Mistral, located between *Hoxa6* and *Hoxa7*, has been reported to recruit WDR5 and the MLL1 complex to activate *Hoxa6* and *Hoxa7* transcription (20). *HOTTIP* is transcribed in an antisense direction from the 5′ end of *Hoxa13*, and is reported to be important for targeting Wdr5 and MLL across *HOXA* and for maintaining 5′ *Hoxa* expression and H3K4me3 in distal tissues (21).

The menin tumor suppressor protein is a common component of MLL1 and 2 complexes, and has been reported to be important for MLL recruitment to target genes and for the regulation of *Hox* expression (22). Menin functions as an adaptor molecule, binding to MLL1while also interacting with the protein Psip1/p75 at a distinct surface. Neither menin nor MLL1 alone can interact with Psip1 (23–25).

**Figure 1.**
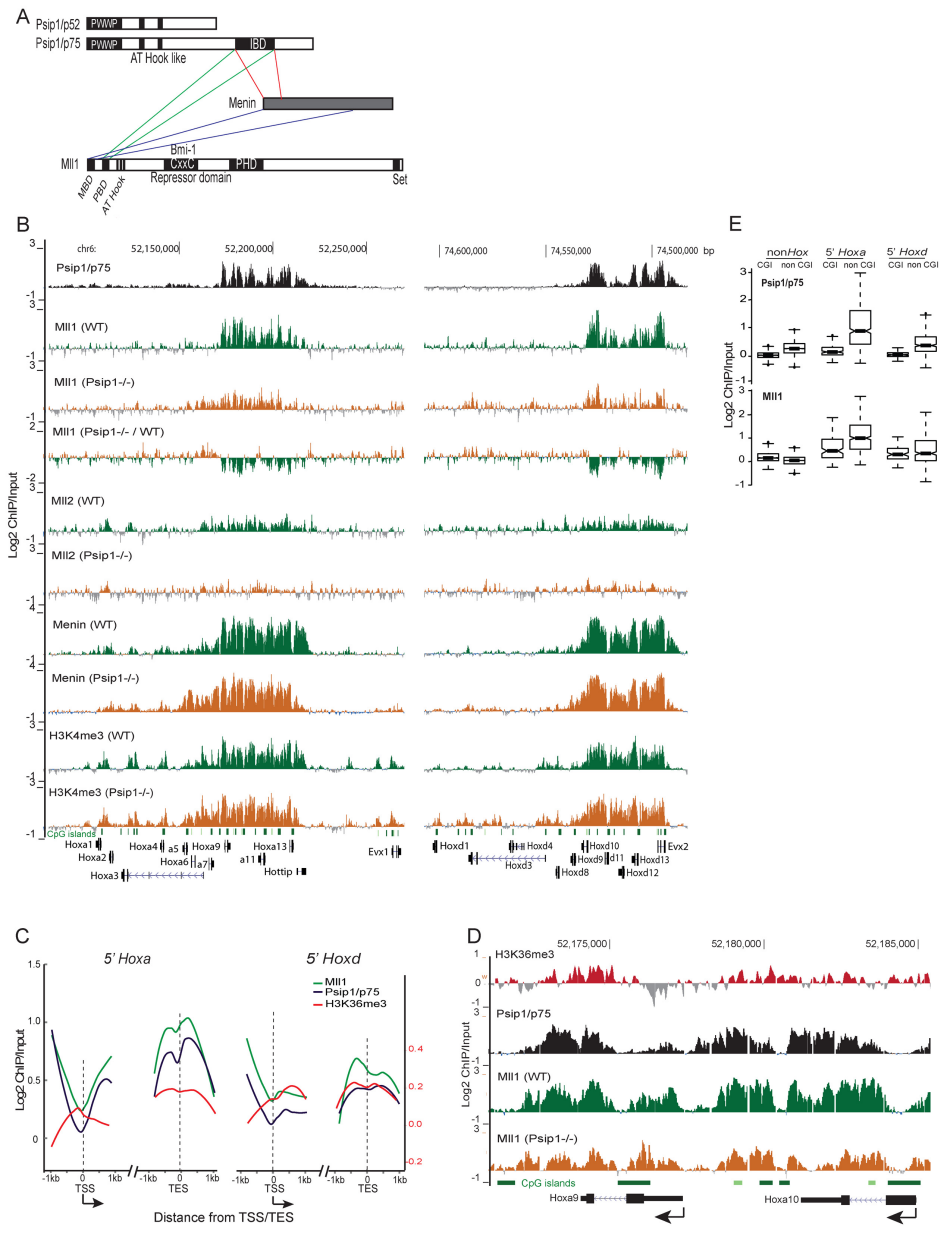
Psip1/p75, Mll1 and menin localization in wild-type and *Psip1*^−/−^ cells. (A) Cartoon of Psip1 p52 and p75 isoforms showing the localization of the PWWP domain, AT hooks and also the integrase binding domain (IBD) domain at the C-terminus of p75 that interacts with menin and Mll1. The menin binding domain (MBD), Psip1 binding domain (PDB), CxxC domain and plant homeodomain (PHD) of Mll are also shown. (B) Mean Log2 ChIP/input for Psip1/p75 Mll1, Mll2, menin and H3K4me3 *in WT and Psip1^−/−^* MEFs over genomic regions encompassing *HoxA* (left) and *HoxD* loci (right). For Mll1, a difference plot for Mll1 ChIP in Psip1^−/−^ versus WT cells is also shown. (n = 2 biological replicates.) Below, the positions of CpG islands and genes are shown. Genome co-ordinates (bp) from the mm9 version of the mouse genome assembly. (C) Averaged Log2 ChIP/input ratios for p75 (black lines), Mll1(green) and H3K36me3 (red) in 2 kb windows surrounding the transcription start site (TSS) or transcription end site (TES) of expressed genes from the 5′ portions of Hoxa and Hoxd. Arrow under TSS indicates the direction of transcription. (D) As in B, but zoomed in view of Psip1/p75, Mll1 and H3K36me3 distributions over Hoxa9 and Hoxa10 in WT MEFS. The Mll1 distribution in Psip1^−/−^ MEFs is also shown. (E) Box plots showing the distribution of Log2 ChIP/input ratios for Psip1/p75 (top) and Mll1 (bottom) over the CpGisland (CGI) and non-CGI portions of expressed non-Hox genes and expressed genes from 5′ *HoxA* and *HoxD*.

PC4 and SF2 interacting protein 1 (Psip1), previously known as LEDGF, is a chromatin protein implicated in; transcriptional regulation—including of *Hox* genes (26), mRNA splicing (27,28), DNA repair (29) and HIV integration (30,31). *Psip1* encodes two isoforms (p52 and p75) which share a common N-terminal PWWP domain that binds to H3K36me3 (28) and is required for MLL1-mediated leukemic transformation (32) (Figure 1A). Psip1 p52, but not p75, interacts with splicing factors and can modulate alternative splicing of weak exons (28). It is the C-terminal domain of p75, absent in p52, which interacts with MLL1 (32) and a variety of other proteins (33–35) (Figure 1A). The mechanism by which Psip1/p75 regulates transcription is not known.

Here, we show that Psip1/p75, rather than p52, is important in regulating *Hoxa* and *Hoxd* gene expression. Psip1/p75 interacts with Mll1, and in *Psip1^−/^*^−^ mouse embryonic fibroblasts (MEFs) Mll1 and Mll2, but not H3K4me3, levels are reduced on expressed *Hoxa* and *Hoxd* genes. Unexpectedly, however, given the assumed association of Mll1 with *Hox* activation, *Hox* genes are up-regulated in the absence of Psip1 and the accompanying loss of Mll1, suggesting that p75, while recruiting Mll1—a supposed activator, acts to repress gene expression. Furthermore, we show that Psip1/p75 is also required to recruit the polycomb group protein Bmi1, and the co-repressor Ctbp1 to expressed *Hox* genes.

This study reveals a potential mechanism through which the p75 isoform of Psip1 regulates the expression of *Hox* genes and it highlights the unexpectedly complex relationship between the polycomb and trithorax machinery. It is clear that these systems cannot simply be considered as opposing repression versus activating protein complexes.

## MATERIALS AND METHODS

### Cell culture

*Psip1^−/−^* and corresponding *WT* immortalized MEFs (30) were a gift of Prof. Alan Engelman. Primary MEFs were derived from 13-day-old (E13) *Psip1^gt/gt^* embryos and their *WT* littermates as described previously (28). *Psip1^−/−^* MEFs were transduced with retroviral vectors containing p52 and p75 cDNAs -pLPX-p52HA and pLPX-p75HA (30) and packaged in PLAT-E cells according to a standard protocol (Clonetech). Transduced cells were selected with 2.5 μg/ml puromycin and stably expressed HA-tagged Psip1 isoforms were detected by immunoblotting with Psip1 antibodies (Bethyl lab. A300–847).

### Lentiviral knockdown

Lentiviral micro RNA (Gift from Dr. Gijsbers, KU Leuven) specifically targeting p75, or both p52 and p75, isoforms of Psip1 were transduced into wild-type (WT) MEFs. Stably transduced MEFs were selected using blasticidin (10 μg/ml). The efficiency of knockdown was validated by immunoblotting with antibodies recognizing Psip1 p52 or p75 (28). Psip1 p75 was also depleted in 10.5 dpc mouse distal posterior limb cells (36) using lenti-viral shRNAs (Sigma Aldrich, TRCN0000012116 and TRCN0000012113) and stably transduced cells were selected using puromycin (3 μg/ml).

### Chromatin immunoprecipitation (ChIP)

ChIP was performed as previously described (28), using antibodies for Psip1/p75 (Bethyl laboratory A300–848), Psip1/p52 (Bethyl laboratory A300–847) Mll1 (Active Motif 61295), BMI1 (Millipore 05–637), Ring1B (MBL D139–3), H3K4me3 (Millipore 07–473), menin (Abcam, ab4452–50), RNA PolII Ser2p (Millipore 04–1571, Clone 3E10), Ctbp1 (Santa Cruz SC-55502), CBX4 (Abcam ab139815) and Mll2 (Abcam ab15962). ChIPed DNA was amplified with WGA2 using the manufacturer's protocol (Sigma Aldrich) and hybridized to custom Hox arrays (28). All ChIP on chip experiments were done with at least two biological replicates (GEO accession number GSE 49182 for platform GPL13276).

Normalization and analysis of microarray data was as described previously (28). For CpG analysis, CpG islands (CGIs) were identified by finding probes with a minimum of 25 bp overlap with CGI found ±1 kb within genes using Galaxy software. CGI positions were taken from the University of California Santa Cruz (UCSC) table browser.

Enrichment analysis for ChIP and run-on data was performed for probes ±1 kb from transcription start sites (TSS) or transcription end site (TES). The smoothed conditional mean plots were generated using the R package ggplot2 and the geom_smooth function.

The following mm9 coordinates were used for quantification of ChIP enrichment; non-expressed 3′ *Hoax*(*Hoxa1* to *Hoxa7*) genes chr6:52,101,011–52,172,728, expressed 5′ *Hoxa* genes (*Hoxa9-Hoxa13*) chr6:52,171,296–52,211,033, 3′ non-expressed *Hoxd* (*Hoxd1* to *Hoxd9* genes) chr2:74,534,258–74,606,421, expressed 5′ *Hoxd* genes (*Hoxd9-Hoxd13*) chr2:74,484,916–74,537,448. To test the significance of differential ChIP enrichment at genomic regions a Wilcoxon rank-sum test was performed with a correction for multiple testing (Holm method) using the R statistical program.

For sequential ChIP (SeqChIP), antibodies were covalently coupled to Dynabeads with antibody coupling kit, (Invitrogen Cat. 14311D), using the manufacture's protocol. The first ChIP was eluted with 10 mM DTT and the elute was diluted 30 times with Radio-Immunoprecipitation Assay (RIPA) (50 mMTris, pH 7.5, 150 mMNaCl, 1% IGEPAL CA-630, 0.5% deoxycholate) buffer before continuing with the second ChIP. Primers used for ChIP qPCR are given in Supplementary Table S1.

### Expression analysis

Expression microarray was performed with four biological replicates of *WT* and*, Psip1^−/^^−^* MEFs, as described previously (37). Gene Ontology (GO; Biological Process) enrichment analysis was performed using the GO enrichment analysis and visualization web tool (GORilla). A False Discovery Rate (FDR) *q*-value cut-off of 0.01 was used to select significantly enriched GO terms.

For reverse transcriptase-polymerase chain reaction (RT-qPCR), cDNAs were prepared with Superscript II (Invitrogen) reverse transcriptase using random primers. The list of specific primers used is given in Supplementary Table S1. RT-qPCR was done with three biological replicates of *WT* and *Psip1^−/−^* MEFs and *Psip1^−/−^*MEFs rescued with p52-HA or p75-HA cDNA on a LightCycler 480 (Roche Diagnostics). Data were normalized to *Gapdh* and the error bars indicate standard error of mean (s.e.m.) from three biological replicates. Similarly, RT-qPCR for *WT* MEFs depleted for Psip1 isoforms was done for three biological replicates.

### Run-on transcription

Approximately 10^7^ MEFs were resuspended in hypotonic buffer (20 mM Hepes-KOH pH 7.9, 10 mMKCl, 1 mM MgCl_2_, 0.5% NP40, 20% Glycerol) and dounced 25 times on ice. The run-on transcription assay was performed as described previously (38). Run-on RNA was reverse transcribed using whole transcriptome amplification (WTA2) kit according to the manufacturer (Sigma Aldrich), and the resultant cDNA was labeled with Cy3 or Cy5 and hybridized to the same custom tiling arrays used for hybridizing ChIP DNA.

### Nuclear extracts and immuno-precipitations

Cells from 14-cm dishes were trypsinized and pelleted, resuspended in 5 ml of ice-cold swelling buffer (10 mM Hepes, pH 7.9, 1.5 mM MgCl_2_, 10 mM KCl, 0.5 mM DTT, and protease inhibitors) for 5 min, and broken open to release nuclei using a pre-chilled Dounce homogenizer (20 strokes with a tight pestle). Nuclei were pelleted by centrifugation at 228 *g* for 5 min at 4°C and resuspended in 1 ml of RIPA buffer and protease inhibitors + Benzonase (Novagen; ﬁnal concentration, 1.25 U/μl) and incubated for 30 min on ice. Extracts were cleared by centrifugation at 15500 *g* for 10 min at 4°C.

Protein A Dynabeads (Invitrogen), were incubated with 5 μg of α HA antibodies (for HA pulldown) or control immunoglobulin G (IgG) antibodies for 1 h in phosphate buffered saline. Equivalent nuclear protein amounts were incubated with the beads coupled to antibodies for 1 h. After 4 wash steps in RIPA buffer, bound proteins were eluted using 2× sodium dodecyl sulphate (SDS) loading buffer, and separated on a NuPAGE gel, blotted to Polyvinylidene fluoride (PVDF) membrane, and immunoblotted with antibodies recognizing; Mll1 (Active Motif 61295, 1:1000), BMI1 (Millipore 05–637, 1:1000), Ring1B (MBL D139–3, 1:2000), EZH2 (BD Biosciences 1:3000) and HA tag (Sigma H6908, 1:1000). For the GFP trap experiment, *WT* MEFs were stably transduced with green fluorescent protein-p75 (GFP-p75) (39), GFP-p75 complex was purified according to the manufacturer's protocol (ChromoTek).

## RESULTS

### Psip1/p75 localizes to expressed *Hox* genes

Surviving Psip1 gene-trap mutant mice show homeotic skeletal transformation phenotypes, similar to those of *Hoxa4, 5* and *6* mutant animals (26). Moreover, knockdown of PSIP1 in human cells showed that mRNAs of 5′*HOXA* genes, but not genes of the other *HOX* loci, are among the most up-regulated (26,40). This suggests that the *HoxA* cluster may be a specific target for regulation by Psip1.

To identify Psip1/p75 occupancy on *Hox* loci we performed ChIP for endogenous p75 from *WT* immortalized MEFs with an antibody (A300–848), whose specificity for Psip1/p75 and inability to recognize the p52 Psip1 isoform we have confirmed previously (28). ChIP'd DNA was hybridized to custom arrays covering all four *Hox* loci and several other developmental genes (28). Psip1/p75 was enriched over *HoxA* and *HoxD* (Figure 1B) but was not detected at *HoxB* or *HoxC* loci in these cells (Supplementary Figure S1A). The preference for p75 occupancy over *Hox* A and D clusters does not reflect an intrinsic property of the DNA sequence there: ChIP in independent primary MEFs derived in our laboratory (28) showed specific occupancy of p75 over *Hoxa, b and c* genes which are expressed in those cells (data not shown), suggesting that Psip1/p75 generally binds to expressed *Hox* genes.

We found that the distribution of Mll1 and menin across *HoxA* and *HoxD* was highly similar to that of p75 (Spearman's correlation between p75 and Mll1 *ρ* = 0.82, *P* < 0.01) (Figure 1B–D) and, like p75, these proteins were also not detected at *HoxB* and *HoxC* clusters in these MEFs (Supplementary Figure S1A). Psip1 p75, Mll1, menin and H3K4me3 were abundant at the 5′ ends of *HoxA* and *HoxD* (*Hoxa9* to *a13*, *Hoxd10* to *d13*) but not at the 3′ end (Figure 1B). Mll1 and p75 were also not enriched at the silent non-*Hox* polycomb-target *Shh* gene (Supplementary Figure S1B and C).

At *Myc*, p75 is present at the 3′ end of this active gene, as has been seen more globally for Psip1/p52 (Supplementary Figure S1B and C), consistent with targeting to H3K36me3 via the PWWP domain common to both Psip1 isoforms (28) (Figure 1C and D). Mll1 and Menin have a broad distribution across *Myc*, suggesting Psip1 independent binding at this locus (Supplementary Figure S1C).

At 5′*Hoxa* and 5′*Hoxd* genes we found that, like p75, Mll1 is partially excluded from CGIs (Figure 1D and E) but is enriched ∼1 kb downstream from the TSSs, over gene bodies particularly near TESs and beyond (Figure 1C and D). This is a similar profile to that of H3K36me3 (Figure 1C and D) (28) and is consistent with previous analysis of Mll1 distribution over late *HOXA* genes in a human lymphoma cell line (41). This suggests that Mll1 recruitment to chromatin may be dependent on its interaction with Psip1 and not binding of its CXXC domain to unmethylated CGIs.

### Psip1 is required for Mll1 recruitment to expressed *HoxA* and *HoxD*

**Figure 2.**
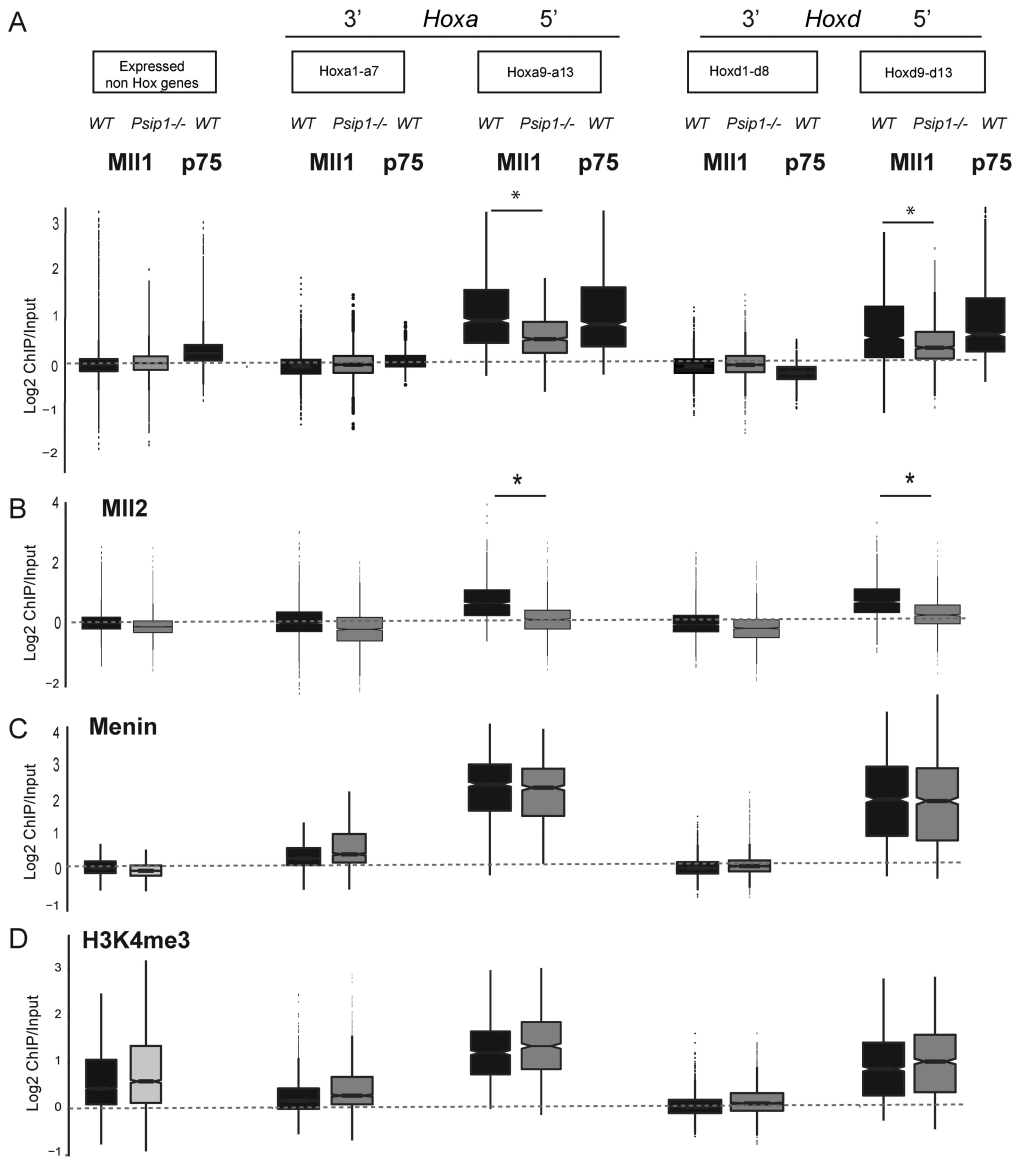
Psip1 loss results in reduced Mll1 and Mll2 at Hox loci. Box plots showing the distribution of; (A) Mll1, Psip1/p75, (B) Mll2 (C) menin and (D) H3K4me3 in *WT* (black boxes) and *Psip1^−/−^* (gray boxes) cells at expressed non-*Hox* genes, 3′ *Hoxa* genes (*Hoxa1-a7*), 5′ *Hoxa* genes (*Hoxa9-a13*), 3′ *Hoxd* genes (*Hoxd1-d9*) and *Hoxd10-d13*. Regions with a statistically significant (*P* < 0.01) difference in binding between *WT* and *Psip1^−/−^* cells as assessed with a Wilcoxon rank-sum test are indicated with an asterisk (*). Box plots showing the distribution of Psip p75 are also shown in (A).

The co-occurrence of Mll1 and Psip1/p75 are consistent with Psip1 being required to tether Mll1 at target genes (32). To test this, we performed ChIP for Mll1, Menin and H3K4me3 in Psip1 homozygous null MEFs (*Psip1^−/−^*) derived from mutant embryos that were littermates to the *WT* controls (28,30). There were not extensive changes in Mll1 or menin binding at non *Hox* genes in the mutant MEFs (Figure 2). However, loss of Psip1 significantly (*P* < 0.01) reduced Mll1 binding across 5′ *Hoxa* (*a9-a13*) - and *Hoxd (d9-d13)* genes (Figures 1B and 2A). Menin levels were not reduced (Figure 2C). Surprisingly, H3K4me3 levels were also not significantly changed at either 5′ *HoxD* or *HoxA* (Figures 1B and 2D) in the absence of *Psip1*, suggesting that Mll1 is dispensable for this histone modification at these sites.

To confirm that loss of Mll1 in *Psip1^−/^*^−^MEFs is not due to differences in cell types, we repeated the Mll1 ChIP in limb mesenchyme cells (36) specifically depleted for Psip1/p75 with a short-hairpin (Sh) RNA. Mll1 was significantly reduced over *Hox* genes in the p75 knockdown cells compared to controls. The p52 isoform of Psip1 was not ablated by the p75 ShRNA (Supplementary Figure S1D), confirming that it is indeed Psip1/p75 that is required for Mll1 targeting.

Both Mll1 and Mll2 are implicated in regulating *Hox* genes (5) and although a direct interaction between p75 and Mll2 has not been demonstrated, a recent report (42) and our GFP-p75 trap data, see below, indicate the presence of Mll2 with p75 complexes, possibly through the common interactor Menin (25). Indeed, we detected Mll2 over both *HoxA* and *D* loci in *WT* MEFs (Figure 1B) and Mll2 levels were significantly reduced over 5′*Hoxa* and *Hoxd* genes in *Psip1^−/−^* MEFs (Figure 2B).

### Absence of Psip1/p75 leads to mis-expression of *Hox* genes

**Figure 3.**
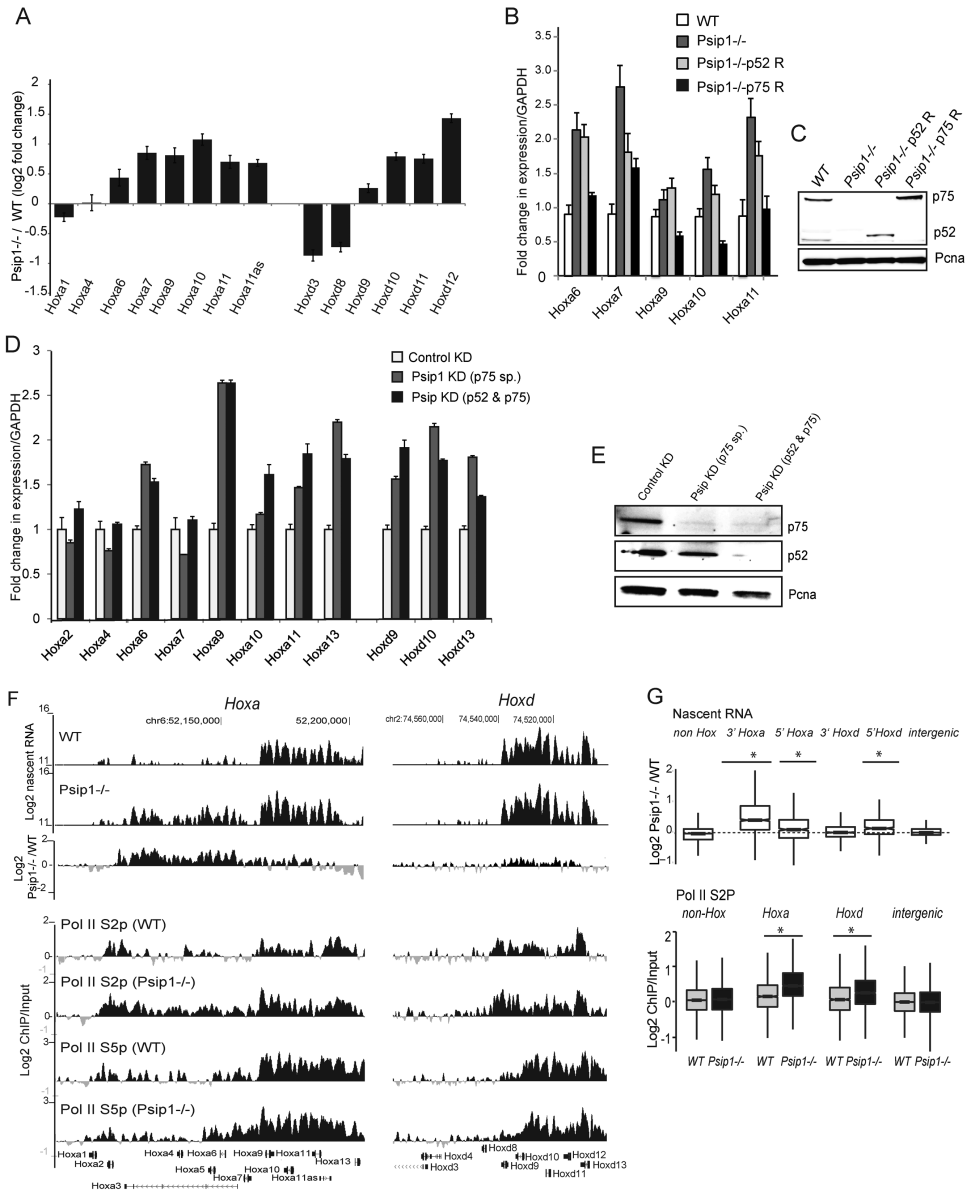
Psip1/p75 represses the expression of *Hox* genes. (A) Log2 fold change in mean (+/- s.e.m) microarray expression levels of *Hoxa* and *Hoxd* genes in *Psip1^−/−^* versus *WT* (*n* = 4 biological replicates each). (B) Mean (+/- s.e.m.) expression of *Hoxa* genes, normalized to *Gapdh*, assayed by RT-qPCR, in *WT* (white) and *Psip1^−/−^* (dark gray) cells, and in Psip1 mutant cells rescued with p52 (light gray) or p75 (black bars) Psip1 isoforms (*n* = 3 biologial replicates). (C) Immunoblot to detect p75 and p52 Psip1 isoforms in *WT*, *Psip1^−/−^*, p52 rescue, and p75 rescue cells. Pcna served as loading control. (D) Mean (+/- s.e.m.) *Hoxa and Hoxd* gene expression, normalized to *Gapdh*, in *WT* MEFs after specific knockdown of p75 isoform (gray bars), or both p52 and p75 isoforms of Psip1 (black bars) and a control scrambled micro-RNA (white bars). (E) Immunoblot to detect p75, p52 and Pcna in cells transfected with control, p75-specific, and p52 + p75 isoform specific lentiviral micro-RNAs. (F) Top: Mean log2 signal of run-on transcribed RNA from *WT* and *Psip1^−/^*^−^ MEFs over *HoxA* (left) and *HoxD* (right), established by hybridization of cDNA from run-on transcripts to custom tiling arrays. (*n* = 3, 2 biological and 1 technical replicates). A difference plot for *Psip1^−/^^−^versus* WT cells is also shown. Bottom: Mean Log2 ChIP/input for Ser2 (S2p) and Ser5 (S5p) phosphorylated PolII in WT and Psip1^−^^/^^−^ MEFs (n = 2 biological replicates). (G) Box plots show: Top—Log2 ratio of Psip1^−/−^/WT run-on transcribed RNA (Nascent RNA) over non Hox genes, genes from the 3′ and 5′ ends of *HoxA* and HoxdD, and intergenic regions. Bottom—Log2 ChIP/input for Ser2 phosporylated RNA Polymerase II (Pol II (S2p) in WT and Psip1^−/−^ MEFs over non-Hox genes, *Hoxa*genes, Hoxd genes and intergenic regions (n = 2 biological replicates). Regions with a statistically significant difference (P < 0.05) in S2p between WT and Psip1^−/−^ cells as assessed by the Wilcoxon rank-sum test are indicated with an asterisk (*).

Microarray analysis shows elevated levels of mRNAs from several 5′ *Hoxa and Hoxd* genes in *Psip1^−/^*^−^ MEFs compared to *WT* cells (Figure 3A) and, surprisingly, given the usual association of Trx proteins with gene activation, this included 5′ *Hoxa* genes (*a9, a10* and *a11*), that lose Mll1 binding in the mutant cells. This was confirmed by quantitative RT-PCR (Figure 3B). GO analysis of genes differentially expressed (FDR *q* < 0.01) between *WT* and *Psip1^−/^*^−^show enrichment of terms associated with anatomical structure morphogenesis and developmental process (Supplementary Figure S2A), consistent with the craniofacial and skeletal abnormalities of surviving *Psip1* mutant mice (26).

To test whether mis-regulation is due to loss of the p52 or p75 Psip1 isoforms, we rescued each isoform by retroviral transduction of appropriate cDNAs into *Psip1^−/−^* MEFs (Figure 3C). Psip1 p75 was able to reverse the elevated mRNA levels of many of the up-regulated *Hoxa* genes, and p52 had modest or no effects on expression of the tested genes, with the exception of *Hoxa7* (Figure 3B).

We further confirmed a direct effect of p75 loss on *Hoxa* mRNAs by knocking down Psip1/p75 protein levels in *WT* MEFs using two different miRNAs, one specific for the p75 isoform and that has no effect on Psip1/p52, and the other targeting the N-terminal domain common to both isoforms (Figure 3E). This resulted in increased expression of *Hoxa9-a13*, and *Hoxd9-d13* mRNAs (Figure 3D). Steady-state levels of mRNAs from 3′ *Hox (a2-a4)* were unaltered, compatible with data from *Psip1^−/−^* cells (Figure 3A and B).

Elevated 5′ *Hoxa* mRNA levels, was also validated in primary MEFs derived from E13.5 *Psip1^gt/gt^* embryos compared to their wild-type littermates (26,28) (Supplementary Figure S2B). Together, these data suggest that Psip1 p75 has a specific role in regulating the expression level of *Hox* genes.

Given our previous demonstration of a role for Psip1 p52 in mRNA processing (28) we determined whether Psip p75 affected the level of transcription *per se*. Run-on transcription in *WT* MEFs detected high nascent RNA synthesis from the 5′ ends of the *HoxA* and *HoxD* clusters, and not from the 3′ regions (Figure 3F). In *Psip1^−/−^* cells, run-on RNA levels were elevated at 5′ *Hoxa* and *Hoxd* genes, but not at non-*Hox* genes or intergenic regions, or at the 3′ end of *HoxD* (Figure 3F and G, Supplementary Figure S2C). Intriguingly, elevated nascent RNA was also detected over part of the 3′ end of *HoxA*, even though there is no Psip1/p75 bound at this region and no change in levels of mature mRNA from *Hoxa4* is detected in *Psip1* mutant cells (Figure 3A and D). However, we note that the extent of this transcription corresponds to the large annotated *Hoxa3* transcript whose TSS is located toward the 5’ of *Hoxa*, between *Hoxa6* and *Hoxa7*. This signal may also arise from unstable, unprocessed RNA. We also noted slightly elevated levels of Menin and H3K4me3 at 3′*HoxA* in the mutant cells though this increase was not statistically significant (Figures 1B and 2C and D).

Our data are consistent with Psip1 playing some role in regulating transcription of *Hox* genes. Furthermore, the enrichment of p75 on gene bodies and toward the TES of transcribed *Hoxa* genes (Figure 1B–D), together with direct binding to H3K36me3 (28), suggests that Psip1 could function to regulate transcription elongation. To investigate this we examined the levels of the elongating serine 2 phosphorylated form of RNA polymerase II (PolII S2p) (43,44) by ChIP in *WT* and *Psip1* mutant cells. The levels of PolII S2p were significantly elevated across *HoxA* and *HoxD* in *Psip1^−/^*^−^ cells (Figure 3F and G). Levels of the serine 5 phosphorylated initiating form of PolII (PolII S5p) were not changed. These data suggests that Psip1/p75 functions to restrain the elongation of transcription from paused/poised Pol II at *Hox* loci and that this may operate through Mll1 retention—at least for 5′ genes of the cluster.

### Absence of Psip1 leads to loss of Bmi1 and Ctbp1

**Figure 4.**
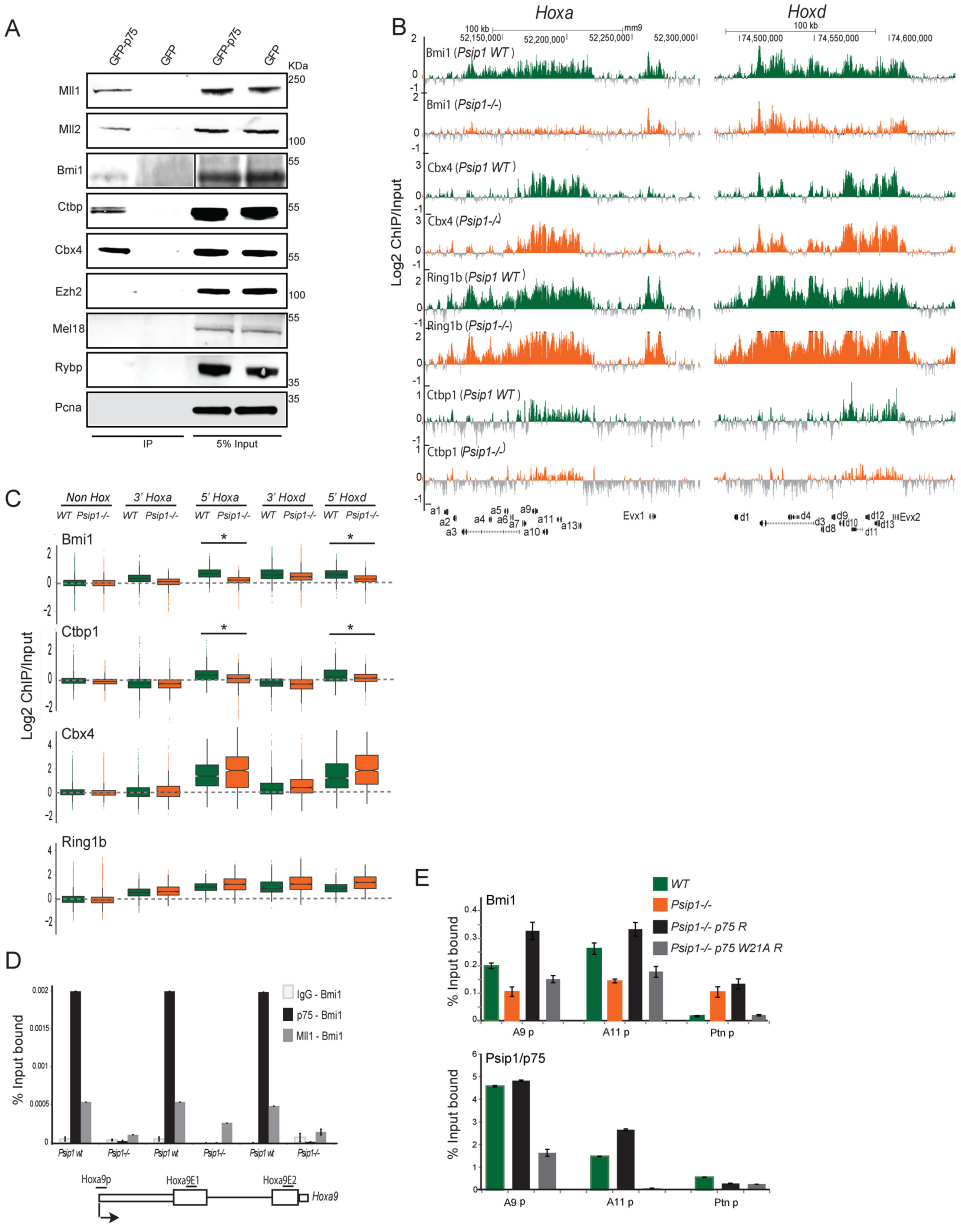
Loss of Psip1 leads to reduced Bmi1 and Ctbp1 on target genes. (A) Psip1/p75 complexes purified from stably transduced GFP-p75, and GFP (control) cells, and immunoblotted using antibodies against Mll1, Mll2, Bmi1, Ctbp1, Cbx4, Ezh2, Mel18, Rybp and Pcna. Note that 5% input extract were also loaded. (B) Mean Log2 ChIP/input for Bmi1, Cbx4, Ring1B and Ctbp1 in *WT* and *Psip1*^−^*^/^*^−^ MEFs *over* *HoxA* (left) and *HoxD* clusters (right) using custom tiling arrays as in Figure 1. (C) Box plots showing Log2 ChIP/input distributions for Bmi1, Ctbp1, Cbx4 and Ring1B in WT and Psip1^−/−^ MEFs over 5′ and 3′ regions of *HoxA* and *HoxD* clusters, and non-Hox genes (n = 2 biological replicates). Asterisk (*) indicates a significant difference in ChIP/input signal between WTand Psip1^−/−^(P < 0.01, Wilcoxon rank-sum test). (D) Sequential-ChIP qPCR over promoter (Hoxa9p), exon1 (Hoxa9E1) and exon 2 of Hoxa9 (Hoxa9E2) in Psip1 WT and Psip1^−/−^ MEFs. First, ChIP was performed with covalently coupled IgG (IgG-Bmi1), p75 (p75-Bmi1) and Mll1 (Mll1-Bmi1), followed by Bmi1 antibodies for second ChIP. Schematic below shows the Hoxa9 gene and primers used for PCR. (E) ChIP qPCR for Bmi1 and p75 in WT (green) and Psip1^−/−^ (orange) MEFS, and inPsip1^−/−^ rescued with WT p75 (Psip1^−/−^p75 R; black) or p75 with W21A PWWP point mutation (Psip1^−/−^p75 W21A R; gray). Ptn promoter primers (Ptnp) were used as a control. Mean (+/- s.e.m.) percent (%) input bound (n = 3) are plotted.

To further investigate the mechanism through which Psip1 and Mll1 restrict expression from *Hox genes*, we performed a GFP trap experiment using stably expressed GFP-p75 in *WT* MEFs. Consistent with previous studies (8,32) and with our ChIP analyses, Mll1 and Mll2 were detected interacting with Psip1. Given the known role of the PRCs in repressing *Hox* gene expression via paused polII (44) and in antagonizing trithorax, we looked for members of the PRC1 and PRC2 complexes in the proteins pulled down with GFP-p75 (Figure 4A). We detected no Ezh2, the HMTase from PRC2, nor did we detect Mel18 from PRC1 or Rybp—a member of a non-canonical PRC1 complex (45–47). However, we did detect the PRC1 components Bmi1 and Cbx4. Interestingly, Ctbp1 a transcriptional co-repressor was also detected in the GFP-p75 trap. Interactions amongst Mll1, Bmi1 and Psip/75, but not p52, were confirmed by pulldown of HA-tagged Psip1 isoforms (Supplementary Figure S3A). Not all PRC1 members interact with Psip1/Mll1 complex—Ring1B, a core member of PRC1 was not detected (Supplementary Figure S3A).

Bmi1, Ctbp1 and Cbx4 have been previously shown to interact directly with the CxxC domain of Mll1 (48). These data prompted us to investigate the occupancy of these, and other polycomb-associated proteins, in *WT* and *Psip1^−/^*^−^ MEFs. Bmi1, and Ring1B were detected at *HoxA* and *D* clusters, even over transcribed genes in *WT* MEFs (Figure 4B). Like Psip1 and Mll1, Cbx4 and Ctbp1 were enriched at the expressed 5′ *Hoxa* and *d* genes and not the 3′ regions of these clusters. The absence of Psip1 in mutant MEFs had no significant effects on Bmi1, Cbx4 and Ring1B levels at non-expressed *HoxB* and *C* (Supplementary Figure S3B), or at non-*Hox* genes (Figure 4C). However, Psip1 absence led to significant loss of Bmi1 and Ctbp1 over 5′ *HoxA* and *D* (Figure 4B and C). We also note that there is some reduction of Bmi1 detected at 3′ *HoxA* and *D* in Psip1 mutant cells but this does not reach statistical significance. Ctbp1 is not present at these regions even in wild-type cells.

To verify co-occupancy of Psip1/Mll1 and Bmi1 over target genes, we performed sequential ChIP, first using covalently coupled IgG, p75 and Mll1 antibodies and then the second ChIPs done with antibodies recognizing Bmi1. In *WT* MEFs, we detected high co-occupancy of p75 with Bmi1, as well as Mll with Bmi1, over the promoter, exon1 and exon2 of *Hoxa9* (Figure 4D). In *Psip1^−/−^* MEFs, p75-Bmi co-occupancy was eliminated and Mll/Bmi1 occupancy greatly reduced (by 50–80%).

To confirm a direct role for Psip1 in Bmi1 retention at 5′ *HoxA* we performed ChIP for Bmi1 and Psip1/p75 in *WT* and *Psip1^−/−^* MEFs, and in *Psip1^−/^^−^* cells rescued with the p75 isoform. qPCR showed restoration of Bmi1 levels over tested *Hoxa* genes after p75 rescue (Figure 4E). A version of p75 in which a critical residue in the PWWP aromatic cage involved in H3K36me3 binding is mutated (W21A) (49) failed to restore Bmi1 binding (Figure 4E) and indeed W21A p75 failed to bind to *Hox* loci, consistent with a role for H3K36me3 recognition in p75 targeting (28).

## DISCUSSION

It is generally assumed that members of polycomb complexes are involved in gene repression and that trithorax group members counteract this and help to maintain an active state (6–8). Here we have demonstrated that Psip1/p75 is required to retain Mll1/2 and Bmi1/Ctbp1 at *HoxA* and *HoxD* loci (Figures 1 and 2), with the net result of dampened gene expression. Contrary to the assumed role of Mll1 in maintaining gene activity, the loss of Mll1 (and Mll2) that occurs in the absence of Psip1 results in the upregulation of *Hoxa* and *Hoxd* gene transcription and mRNA levels. These data that we obtained in mouse embryonic fibroblasts are consistent with the upregulation of *Hox* genes that was reported after knockdown of PSIP1 in human 293 cells (26,40).

### Psip1, Mll and menin

Menin has been reported to be important for MLL1/2 recruitment to target genes and for *Hox* gene regulation (22). Menin was suggested to bind to MLL1 and to interact with Psip1/p75 using distinct interaction surfaces (23–25) (32). Levels of Menin were not significantly changed in *Psip1^−/−^* cells suggesting that Psip1 is not required for menin targeting (Figure 2).

In MEFs we found similar genomic binding profiles for Psip1/p75, Mll1 and Mll2 (Figure 1) and in the absence of Psip1, Mll1/2 levels were reduced over expressed (5′) genes of the *HoxA* and *HoxD* clusters. However, we found no corresponding loss of H3K4 trimethylation suggesting that Mll1/2 are dispensable for H3K4me3 at these loci. Our data are consistent with persistence of H3K4me3 over some *Hox* genes in both *Mll1^−/^*^−^ and *Mll2^−/^*^−^ ES cells (18) and in *Mll1^−/^*^−^ MEFs (5) and the suggestion that multiple H3K4 HMTs, including SET1, can be found co-bound at the same active genes (18).

### Psip1 and *Hoxa* gene expression

PSIP1, and particularly its PWWP domain, is known to be required for MLL1-mediated leukemogenesis, and for targeting MLL1 fusion partners leading to uncontrolled expression of *HOXA9* in leukemia (32). Similarly, *Psip1* gene-trap mutant (*Psip1^gt/gt^*) mice have posterior skeletal transformations (26), similar to mice with mutation of *Hoxa* genes (50–52).

Here, we have demonstrated specific up-regulation of mRNA expression from 5′ genes of the *HoxA* and *HoxD* clusters (Figure 3) in the absence of Psip1 and have shown that this is dependent on the long p75 isoform of Psip1 and not p52—which we have previously demonstrated interacts with splicing factors to modulate alternative splicing (28). Consistent with recognition of H3K36me3 by the PWWP domain of Psip1 (28,49), p75 is distributed away from CGIs and toward the 3′ end of *Hox* genes (Figure 1). We show that the Psip1 PWWP domain is required for Bmi1 recruitment to 5′ *HoxA* and *HoxD* (Figure 4).

### Psip1 and the interplay between polycomb and trithorax regulation

Our results suggest that Psip1/p75 functions to recruit Mll, and yet to restrain expression, from specific *Hox* loci. Clues to the mechanism underlying this may arise from our observation that Psip1 is also needed to recruit the repressors Bmi1 and Ctbp1 (Figure 4). We therefore suggest that at *HoxA* and *HoxD*, Psip1 tunes gene expression through its ability to recruit both Mll1 and Bmi1/Ctbp1.

Mll1 is a large multidomain protein that, by binding different proteins, can act either as a transcriptional activator or a repressor. Bmi1 has been reported to bind to the repressive (CxxC) domain of Mll1 (48) and we have confirmed that Bmi1 is present in chromatin that also contains Mll1 (Figure 4). Supporting a functional link between Psip1 and Bmi1, the up-regulation of 5′ *Hoxa* genes in *Psip1^−/−^* cells is similar to that seen in *Bmi1^−/−^* MEFs (53) and *Psip1* gene-trap mutant (*Psip1^gt/gt^*) mice have posterior skeletal transformations (26) similar to mice with mutation of Bmi1 (54).

The HMTase activity of Mll1 is known to be dispensable for its essential functions (16). Since we show that H3K4me3 levels are not altered over 5′*Hoxa* genes in the absence of Psip1 and loss of Mll1 binding (Figures 1 and 2), we suggest that the Psip1-Mll1 interaction serves to recruit repressors, including the Bmi1 component of PRC1 and the co-repressor Ctbp1. Ctbp1 has been reported to colocalize with Bmi1 in the nucleus (55) and mutation of Ctbp1 in flies leads to loss of polycomb group protein recruitment to polycomb response elements (PREs) (56). Similarly, the increased expression of *Hoxd* genes reported in *Mll1^−/^^−^* MEFs (5) could be due to loss of repressors, such as Bmi1 and Ctbp1. Psip1 has been shown to promote homologous recombination (HR) by interacting with CtIP (29), CtIP interacts with CtBP1 and both proteins are components of the RBP-Jκ/SHARP corepressor complex (57). Recently, a role for H3K36me3 and Psip1 in promoting HR at transcriptionally active loci has been demonstrated (58), which is consistent with the recruitment of Psip1 to expressed gene bodies marked by H3K36me3 (28).

We have identified a new Psip1-dependent pathway of control of *Hox* loci that involves both repressors (Bmi1, Ctbp1) and proteins traditionally thought to be involved in maintaining gene activation (Mll1/2). This appears to operate, at least partially, at the level of transcription elongation (Figure 3), but we do not exclude that there may be effects at other levels of RNA processing as well. The polycomb and trithorax systems are usually considered to be antagonistic repressive and activating protein complexes. However, the colocalization of both Trx and polycomb at specific sites on Drosophila polytene chromosomes hints at an interaction between the two systems (59). Other studies have also indicated the presence of repressor complexes at active loci and their role in ‘fine-tuning’ of gene activation (60). Occurrence of proteins known to be implicated with gene repression—e.g. Bmi1, Ctbp1, CBX4 and Ring1B over the expressed *Hox* genes (Figure 4), and up-regulation of mRNAs from Hox genes with the loss of some of these proteins, suggests a more nuanced role of these proteins in fine tuning gene expression.

Finally, our data suggests a new pathway of gene control that may be important for the dysregulation of *HOXA* genes in leukemia (61). MLL is required to maintain *HOXA9* expression in haematopoietic progenitor cells, but then is also required to later repress HOXA9 during the later stages of differentiation (62). Moreover, acute and chronic myeloid leukemias and myelodysplastic syndrome have been associated with fusion of PSIP1 to NUP-98 (63–65).

## SUPPLEMENTARY DATA

Supplementary Data are available at NAR Online.

SUPPLEMENTARY DATA
