# Supplementary material for: Psip1/Ledgf p75 restrains Hox gene expression by recruiting both trithorax and polycomb group proteins
Source: Nucleic Acids Res. 2014 Jul 23;42(14):9021–32. doi: 10.1093/nar/gku647 (PMC4132756; doi:10.1093/nar/gku647)
Supplement: SUPPLEMENTARY DATA [file supp_42_14_9021__index.html]

Psip1/Ledgf p75 restrains Hox gene expression by recruiting both trithorax and polycomb group proteins — Psip1/Ledgf p75 restrains Hox gene expression by recruiting both trithorax and polycomb group proteins — SUPPLEMENTARY DATA 

# Psip1/Ledgf p75 restrains *Hox* gene expression by recruiting both trithorax and polycomb group proteins

## SUPPLEMENTARY DATA

**Files in this Data Supplement:**

- SUPPLEMENTARY DATA
