# Supplementary material for: Psip1/Ledgf p75 restrains Hox gene expression by recruiting both trithorax and polycomb group proteins
Source: Nucleic Acids Res. 2014 Jul 23;42(14):9021–32. doi: 10.1093/nar/gku647 (PMC4132756; doi:10.1093/nar/gku647)
Supplement: SUPPLEMENTARY DATA [file supp_gku647_nar-01056-x-2014-File006.pdf]

Figure S1: Relates to Figures 1 and 2

A) Log2 ChIP/input profiles for Psip1/p75, Mll1, Menin and H3K4me3 across *Hoxb* and *Hoxc* clusters in WT and *Psip1*$^{-/-}$ MEFs. A difference plot for Mll1 in *Psip1*$^{-/-}$ vs WT cells is also shown

B and C) As in Figure 1B but across the *Myc* and *Shh/Rnf32* loci.

D) Box plots showing the level of Mll1 at *Hoxa, b, c, d* genes and non *Hox* genes represented on the microarray. Regions with a statistically significant ($p<0.01$) difference in binding between control knockdown (Control Sh, gray boxes) and Psip1/p75 knockdown (p75 KD, white boxes) cells are indicated with an asterisk (*). Knockdown efficiency of p75 using two shRNAs was validated by immunoblotting (Right) with Psip1. β-actin served as a loading control.

Figure S2:

A) Gene ontology (GO) terms enriched (Log2 p value) at genes differentially expressed ($p<0.01$) between WT and *Psip1*$^{-/-}$ MEFs.

B) Mean (+/- s.e.m., n=3) expression of several *Hoxa, Hoxb* and *Hoxc* genes normalized to Gapdh assayed by RT-qPCR, in *Psip1*$^{gt/gt}$ primary MEFs (black bars) relative to WT MEFs derived from same litter mate embryos (gray bars) (Sutherland et al 2007; Pradeepa et al. 2012).

C) Graphs showing Log2 signal for runon RNA (similar to Figure 3F and G) in *WT* (grey lines) and *Psip1*$^{-/-}$ (black lines) MEFS in 1000bp windows surrounding the transcription start site (TSS) or transcription end site (TES) of expressed non-*Hox* genes and non-expressed genes from the 3' portions of *Hoxa* and *Hoxd* (top), and expressed genes from 5' portion of *Hoxa* and *Hoxd* (bottom). Arrows under TSS indicates the direction of transcription.

Figure S3: Relates to Figure 4

A) HA Pulldown experiment: Empty vector, HA-p52 and HA-p75 immunoprecipitates and 5% input (vector transfected) were immunoblotted with Mll1, Bmi1, Ring1B, Ezh2 and HA tag antibodies.

B) Mean Log2 ChIP/input for Bmi1, Ctbp1, Cbx4 and Ring1B in WT and *Psip1*$^{-/-}$ MEFs over *Hoxb* (left), *Hoxc* (middle) clusters, Olig2 and Myc (right) loci from custom tiling arrays.

References

Pradeepa, M.M., Sutherland, H.G., Ule, J., Grimes, G.R. and Bickmore, W.A. (2012) Psip1/Ledgf p52 Binds Methylated Histone H3K36 and Splicing Factors and Contributes to the Regulation of Alternative Splicing. *PLoS Genet*, **8**, e1002717.

Sutherland, H.G., Newton, K., Brownstein, D.G., Holmes, M.C., Kress, C., Semple, C.A. and Bickmore, W.A. (2006) Disruption of Ledgf/Psip1 results in perinatal mortality and homeotic skeletal transformations. *Mol. Cell. Biol.*, **26**, 7201–7210.

Table S1: Relates to Figure 3 and Figure 4,

List of primers used for qPCR

	Forward	Reverse
Hoxa1	TCCAGCGCAGACCTTTGACTG	GCTCTGTGAGCTGCTTGGTG
Hoxa2	CTGCCTCGGCCACAAAGAATCC	AGCTGAGTGTTGGTGTACGCG
Hoxa4	ACCCCTGGATGAAGAAGATCCAC	TCGCATCTTGGTGTTGGGAAGTT
Hoxa5	CCGCTCAGCCCCAGATCTACC	GTCAGGTAGCGGTTGAAGTGGA
Hoxa6	CACCGACCGGAAGTACACAAG	TGTCTGGTAGCGCGTGTAGGTC
HoxA7	AAGCCAGTTTCCGCATCTACC	GTAGCGGTTGAAATGGAATTCC
Hoxa9	AGAAAAACAACCCAGCGAAG	CAGTTCCAGCGTCTGGTGT
Hoxa10	GTGTCAAGTCCTGAATGGGC	AGAGAAACCAGGCCTGGACT
Hoxa11	CACTGAGGACAAGGCCGGTG	TTGAGCATGCGGGACAGTTG
Hoxa13	CACCTCTGGAAGTCCACTCT	CTCTCAGAGAGGTTTGTCGTGG
Hoxd10	GTGCAGGAGAAGGAAAGCAAAG	TAACGCTCTTACTGATCTCTAGGC
Hoxd13	AGTCCTGGACGCTAGCCAACG	GTAGACGCACATGTCCGGCTG
Hoxb2	TCCGAGGTCGGATCACCATCAG	CTGGAACCAGACTTTGACCTGC
Hoxb4	CTGGATGCGCAAAGTTCACGTG	GCGTCAGGTAGCGATTGTAGTGA
Hoxb7	CGGCCGAGAGTAACTTCCGGA	GTGTGCGCGATCTCGATGCG
Hoxb9	AGGAAGCGAGGACAAAGAGAGG	CTTGTCTCTCACTCAGATTGAG
Hoxc5	TGAACCCTGGGATGTACAGT	TAACTGGTTCGGGACCGCTT
Hoxc6	GACCAGAAAGCCAGTATCCAGA	CTTTTCCTCTTTTCCGCCCA
**ChIP qPCR Primers**		
Hoxa9p	GGAGGGAGGGGAGTAACAAAA	GGAAAATCGCCAGTCAACATC
Hoxa9Exon1	ATCACCACCACCACCCCTAC	CAGGGACAAAGTGTGAGTGTCA
Hoxa9Exon2	GATGCCCCTACACAAAACACC	GCTCGGTCCTTGTTGATTTTC
Olig2 pro	GCCTGACGCTACAGTGACAA	GGCTAATTCCGCTCAATGAA
Hoxa10 pro	ATGTTTGAGGCCGTACTGGT	CTTCCCAAAGTGGCTGGTAA

Hoxa10 E2	TTGCTTGCCTGGCTAAAATC	ACGAAAATCCACCAGTTCCA
Hoxa11 p	AGCCCAATGATGGATTTTGA	GAAGGGAGGCTGGAGAAATC
Ptnp	AGAGAAGAAGCAGGCTGTGC	GGGTGGGTGCTAAGAACAAA

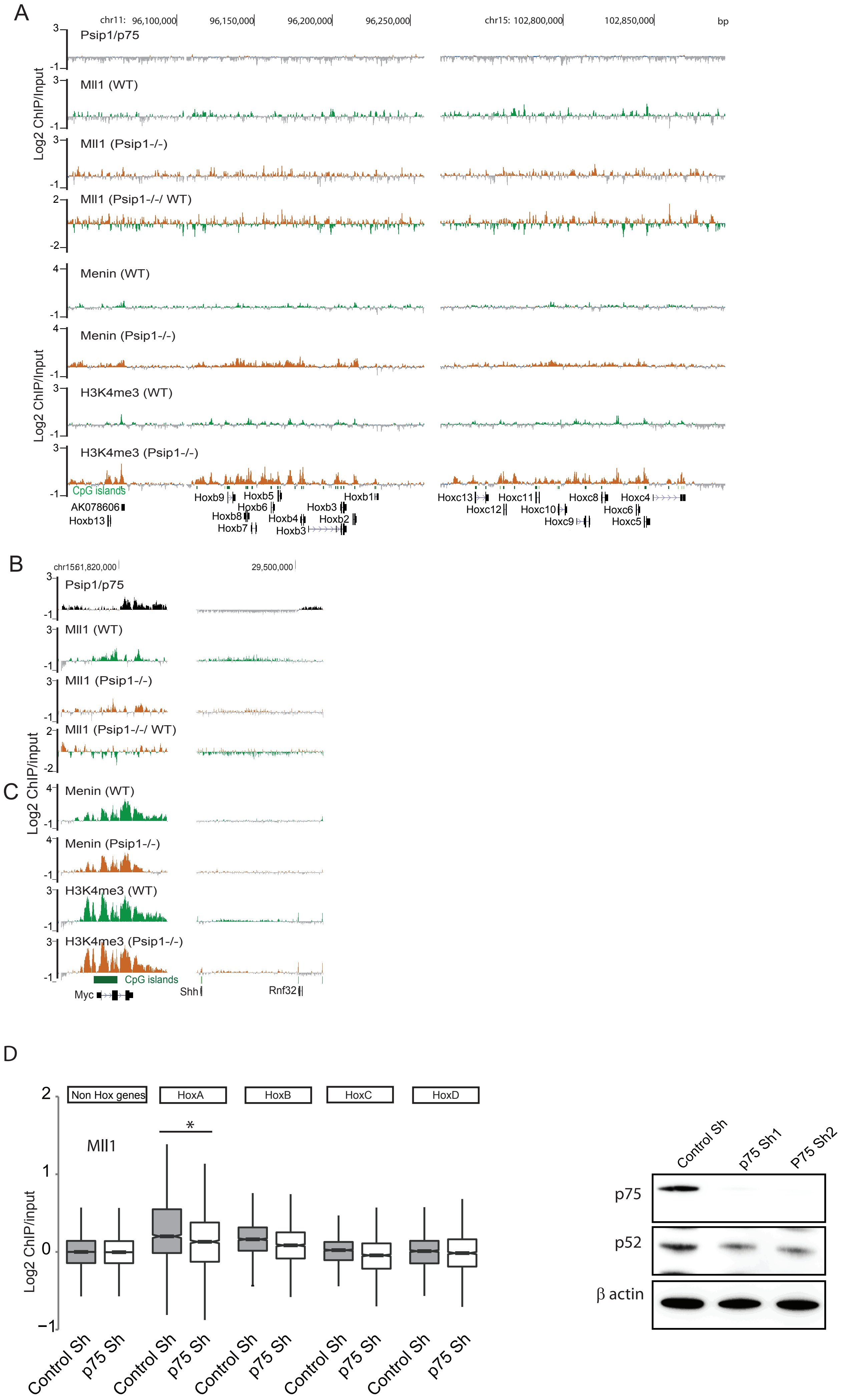

Supplimentary Figure 1

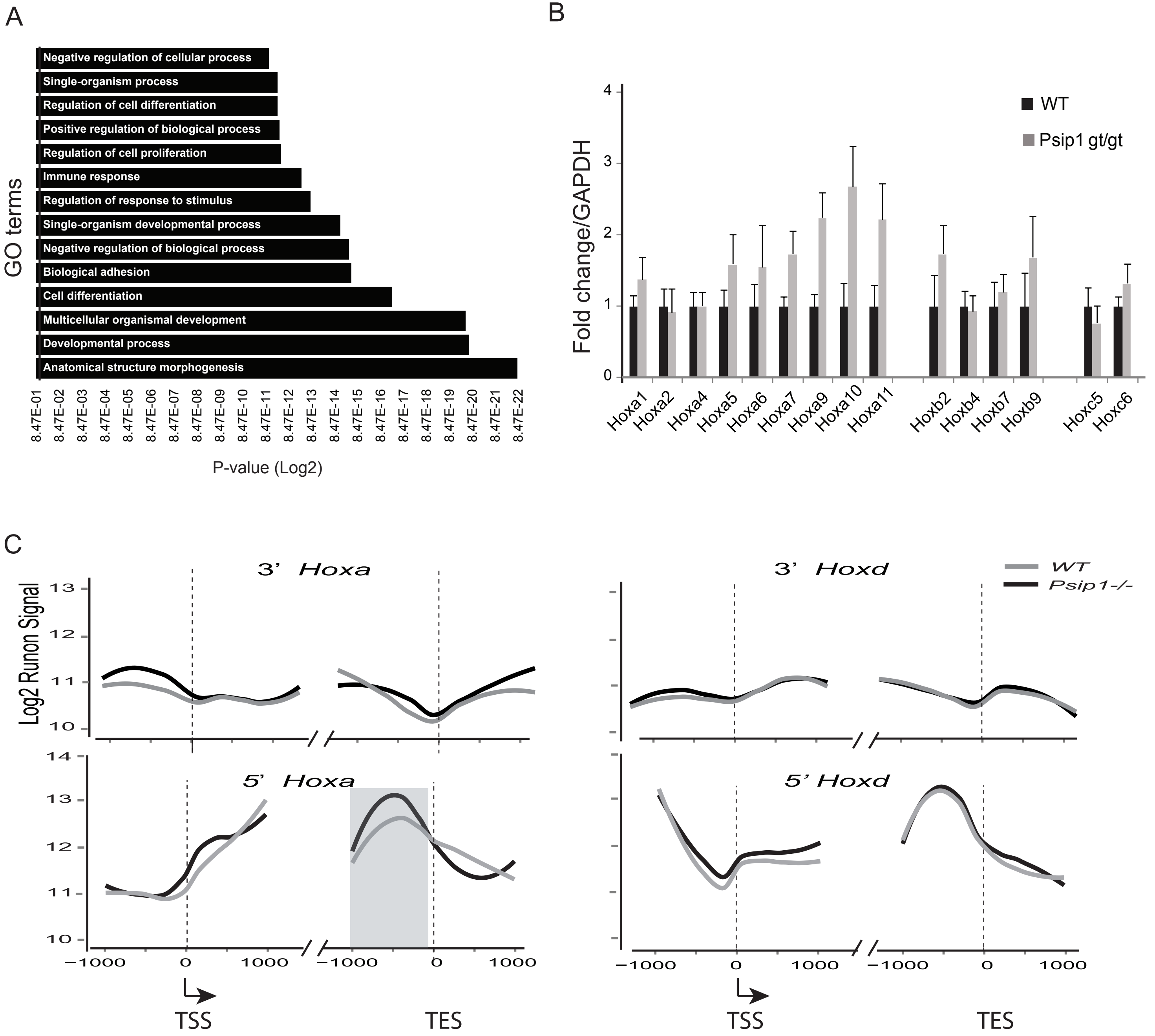

Supplimentary Figure 2

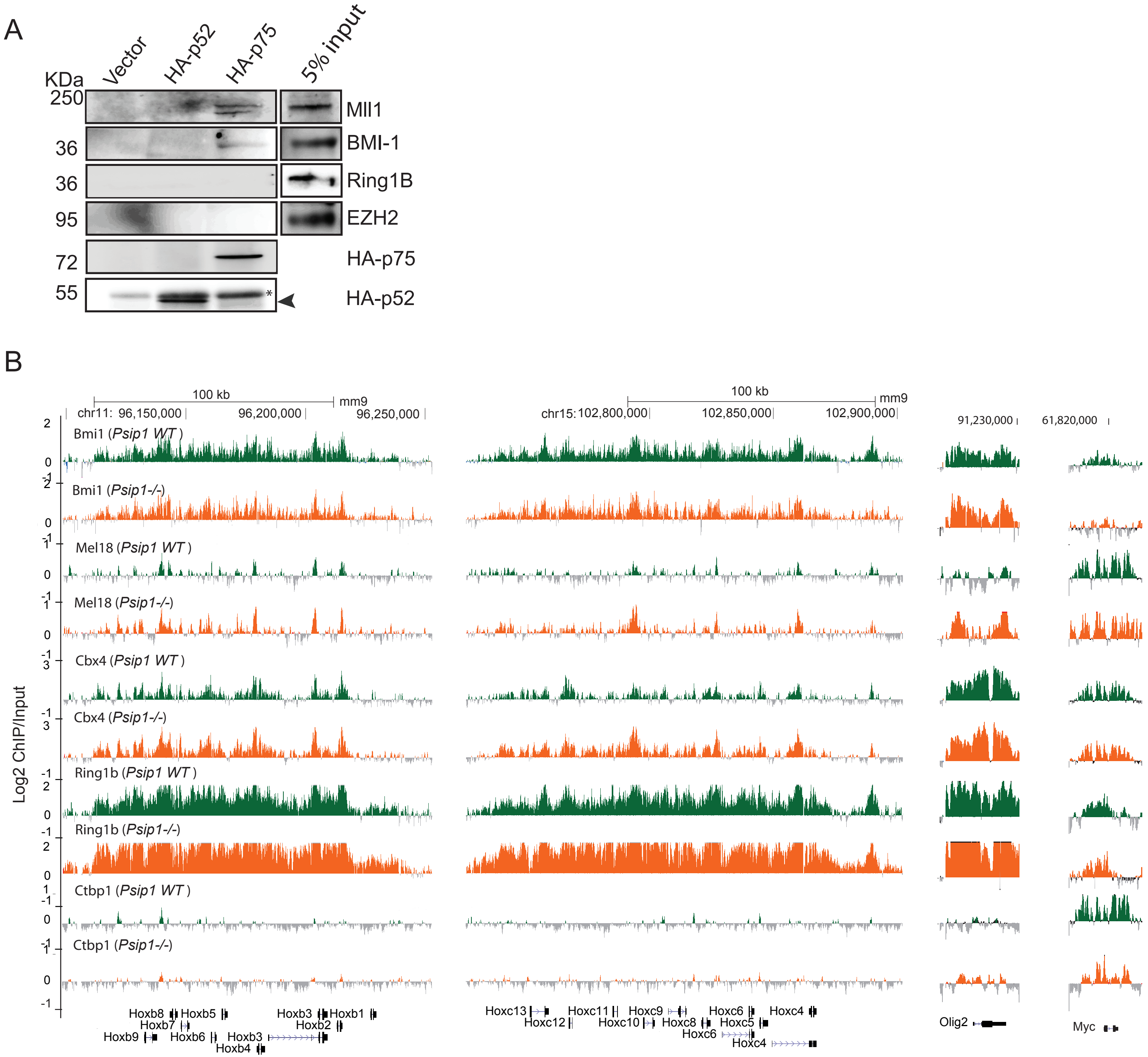

Supplementary Figure 3
